# Combined Treatment with a WNT Inhibitor and the NSAID Sulindac Reduces Colon Adenoma Burden in Mice with Truncated APC

**DOI:** 10.1158/2767-9764.CRC-21-0105

**Published:** 2022-02-02

**Authors:** Maree C. Faux, Janet Weinstock, Sophia Gogos, Emma Prato, Alexander I. Azimpour, Ryan O'Keefe, Yasmin Cathcart-King, Alexandra L. Garnham, Matthias Ernst, Adele Preaudet, Michael Christie, Tracy L. Putoczki, Michael Buchert, Antony W. Burgess

**Affiliations:** 1 Personalised Oncology Division, The Walter and Eliza Hall Institute of Medical Research, Parkville, Victoria, Australia.; 2 Department of Medical Biology, University of Melbourne, Parkville, Victoria, Australia.; 3 Department of Surgery, RMH, University of Melbourne, Parkville, Victoria, Australia.; 4 Olivia Newton-John Cancer Research Institute, Heidelberg, Victoria, Australia.; 5 School of Cancer Medicine, La Trobe University, Bundoora, Victoria, Australia.; 6 Department of Pathology, Royal Melbourne Hospital, Parkville, Victoria, Australia.; † Deceased.

## Abstract

Adenomatous polyposis coli (APC) truncations occur in many colorectal cancers and are often associated with immune infiltration. The aim of this study was to determine whether a combination of Wnt inhibition with anti-inflammatory (sulindac) and/or proapototic (ABT263) drugs can reduce colon adenomas. *Apc*^min/+^ and doublecortin-like kinase 1 (*Dclk1*)^Cre/+^*;Apc*^fl/fl^ mice were exposed to dextran sulphate sodium (DSS) in their drinking water to promote the formation of colon adenomas. Mice were then treated with either a Wnt-signaling antagonist pyrvinium pamoate (PP), an anti-inflammatory agent sulindac or proapoptotic compound ABT263 or a combination of PP+ABT263, or PP+sulindac. Colon adenoma frequency, size, and T-cell abundance were measured. DSS treatment resulted in significant increases in colon adenoma number (*P* < 0.001, *n* > 5) and burden in *Apc*^min/+^ (*P* < 0.01, *n* > 5) and *Dclk1*^Cre/+^*;Apc*^fl/fl^ (*P* < 0.02, *n* > 5) mice. There was no effect on adenomas following treatment with PP in combination with ABT263. Adenoma number and burden were reduced with PP+sulindac treatment in *Dclk1*^Cre/+^;*Apc*^fl/fl^ mice (*P* < 0.01, *n* > 17) and in *Apc*^min/+^ mice (*P* < 0.001, *n* > 7) treated with sulindac or PP+sulindac with no detectable toxicity. PP treatment of *Apc*^min/+^ mice increased the frequency of CD3^+^ cells in the adenomas. The combination of Wnt pathway inhibition with sulindac was more effective in *Dclk1*^Cre/+^;*Apc*^fl/fl^ mice and provides an opportunity for killing *Apc*-mutant colon adenoma cells, indicating a strategy for both colorectal cancer prevention and potential new treatments for patients with advanced colorectal cancer. Outcomes from the results of this study may be translatable to the clinic for management of FAP and other patients with a high risk of developing colorectal cancer.

**Significance:**

Colorectal cancer is one of the most common cancers worldwide with limited therapeutic options. APC and other Wnt signaling mutations occur in the majority of colorectal cancers but there are currently no Wnt inhibitors in the clinic. The combination of Wnt pathway inhibition with sulindac provides an opportunity for killing *Apc*-mutant colon adenoma cells and suggests a strategy for colorectal cancer prevention and new treatments for patients with advanced colorectal cancer.

## Introduction

Many colorectal cancers are initiated by mutations in the adenomatous polyposis coli (*APC*) tumor suppressor gene (1–3). The combination of changes to Wnt signaling and inflammation leads to colon adenomas (4). Adenomas can subsequently develop further mutations which result in progression to adenocarcinoma and malignant disease (5, 6).

A colorectal cancer chemoprevention strategy targeting colon stem cells with *APC* mutations at the same time as inducing apoptosis and/or reducing inflammation has the potential to reduce the impact of this disease. By using *Apc*-mutant mouse models of colon adenoma [*Apc*^min/+^ (7, 8) and *Apc*^fl^ (9, 10)] it should be possible to test the efficacy of drug combinations on the initiation and growth of these adenomas. Because APC truncation underlies most colorectal cancer, drug combinations capable of killing APC-mutant adenoma cells should also be relevant for the treatment of advanced patients with colorectal cancer.

In this article, we measure the effects of pyrvinium pamoate (PP; a Wnt signaling antagonist; ref. 11) combined with either a proapoptotic drug (ABT263; ref. 12) or an anti-inflammatory drug (sulindac; refs. 13, 14) on colon adenoma formation in two independent *Apc*-mutant dextran sulphate sodium (DSS)-treated mouse models (10, 15). These *in vivo* studies are essential for assessing the potential therapeutic efficacy of these drug combinations.

## Materials and Methods

### Mice

All animal procedures were approved by the Animal Ethics Committee of the Walter and Eliza Hall Institute of Medical Research (AEC Project 2018.024; Parkville, Victoria, Australia; ref. 16). The *Apc*^min/+^ and *Apc*^fl/fl^ mice are carried on a C57Bl/6 genetic background and have been described previously (8, 9, 17). Tg(*Dclk1-CreERT2*) BAC transgenic mice were generated (see below) and crossed with *Apc*^fl/fl^ mice [Tg(*Dclk1-CreERT2*);*Apc*^fl/fl^, referred herein to as doublecortin-like kinase 1 (*Dclk1*)^Cre/+^*;Apc*^fl/fl^] on a C57Bl/6 genetic background.

### Generation of the Transgenic *Dclk1-CreERT2* BAC

To generate the *Dclk1-CreERT2* BAC transgene, we used a BAC of the clone RP23-351D24 (∼215 kB, RPCI-23 BAC library, obtained from AGRF) which harbors the *Dclk1* gene. We also used the open reading frame of the tamoxifen-inducible CreERT2 recombinase (18) from the plasmid *GL4.23* (Promega). We employed conventional cloning techniques to construct a polyadenylation (pA)-signal containing *CreERT2*-pA expression cassette with a 5′-homology arm (135 bp) corresponding to the genomic sequence immediately upstream of the ATG start codon of the endogenous *Dclk1* gene. Likewise, this cassette also contained a homology arm at their 3′-end, which corresponded to the target region further downstream in the *Dclk1* gene after exon 3. The latter homology region was preceded by an ampicillin resistance marker flanked in a transgene-specific manner by *frt* recombination sites. We used a recombinase-mediated DNA approach (19, 20) to insert the resulting *Dclk1-CreERT2* expression cassette into the RP23-351D24 BAC in EL250 *E. coli* hosts. The EL250 hosts harbor the λ prophage recombinase system to enable recombination between the BAC and the transgene (20). To identify bacteria harboring a successfully recombined BAC, the cells were grown at 37°C on selective agar (12.5 µg/µL chloramphenicol, 50 µg/µL ampicillin) and clones were confirmed by PCR using transgene-specific primers. We subsequently excised the ampicillin resistance cassette through induction of the Flp-recombinase, which is expressed in response to bacterial growth in medium containing l-arabinose (10% w/v; Sigma). Serial dilutions of the resulting EL250 culture were then plated on selective agar (12.5 µg/µL chloramphenicol) and single colonies were amplified for isolation of the resulting *Dclk1-CreERT2* BAC and PCR confirmation for the excised ampicillin resistance gene. We used PCR analysis to confirm all recombination events in the final *Dclk1-CreERT2* BAC, including the removal of the selection markers, and we also determined the nucleotide sequence of the regions containing the ends of the homology arms and the transgene encoding sequences.

#### Purification and Pronuclear Microinjection of the *Dclk1-CreERT2* BAC

To separate the BAC from its vector backbone, the validated *Dclk1-CreERT2* BAC DNA (80 µg) was digested with *Not*I. The linearized BAC was then applied to a Sepharose CL-4B (Sigma) column that had been preequilibrated with injection buffer (10 mmol/L Tris/HCl pH 7.5, 0.1 mmol/L EDTA, 100 mmol/L NaCl). Fractions of 200–300 µL were collected and analyzed by pulsed field gel electrophoresis to confirm BAC integrity. A fraction containing only linearized BAC DNA at a concentration of approximately 0.2 µg/mL was used for pronuclear microinjection in CBB6F1-derived one-cell embryos as described previously (21). Injected zygotes were incubated in M16 medium overnight and were transferred to the right oviduct of an anesthetized, 0.5-day pseudopregnant outbred CD1 mouse. Genotyping of transgenic founder mice and their litter was performed by diagnostic PCR (see below).

Genomic DNA was extracted from mouse tail biopsies or resected stomach tissue following digestion in 750 µL of DNA isolation buffer (0.05 mol/L Tris/HCl pH 8.0, 0.1 mol/L NaCl, 0.1 mol/L EDTA, 1% SDS) containing 10 µL Proteinase K (10 mg/mL; Sigma).

PCR reactions were carried out in a Dyad Thermal Cycler (Bio-Rad) over 40 cycles (95°C/60 seconds, 53°C/30 seconds, 72°C/60 seconds) with an initial denaturation step at 95°C/5 minutes and a final extension step at 72°C/10 minutes. Each PCR reaction contained 2 µL 10× Taq buffer (Promega), 0.4 µL 50 mmol/L MgCl_2_ (Promega), 0.2 µL 10 mmol/L dNTPs (Roche), 1 µL of each primer (10 µmol/L; Genesearch), 2 µL genomic DNA, 0.2 µL Taq polymerase (Promega) and sterile water up to a total volume of 20 µL. PCR products were analyzed by agarose gel electrophoresis. Expected size of PCR product is 528 bp (forward primer: 5′-TACATCACCAGTGTTTAAACTCA-3′; reverse primer: 5′-TGATGGAGGACATGGACGTTCG-3′).

To ascertain that the *Dclk1-CreERT2* BAC transgene was faithfully labeling *Dclk1*^+^ cells, we crossed transgenic mice with Rosa-LacZ reporter mice (22). Cre-mediated recombination was induced by administration of 3 mg tamoxifen/mouse for 2 consecutive days to compound mutant *Dclk1*^Cre/^;*Rosa*^LacZ/+^ mice [tamoxifen Sigma T5648-5G; 75 mg/mL 10% (v/v) ethanol, 90% (v/v) peanut oil]. Two days later, colonic tissue was harvested and stained with 1/mg/mL X-Gal (5-Bromo-4-chloro-3-indolyl-β-D-galactopyranoside, Sigma-Aldrich B4252; 50 mg/mL in dimethylformamide) overnight at room temperature, as described previously (ref. 23; Supplementary Fig. S1).

### Mouse Treatments

To increase the frequency of colon adenomas, *Apc*^min/+^ mice were provided drinking water containing 2.5% DSS (US Biological MW∼40,000 237292 lot no. 117052559 C17052559) *ad libitum* for 5 days (15, 24). Cre-mediated recombination of *Apc*^fl/fl^ in *Dclk1*^+^ cells was induced by treatment *of Dclk1*^Cre/+^*;Apc*^fl/fl^ with 6 mg of tamoxifen/mouse/day for 3 days (days 1, 3, 5). On day 19, tamoxifen-treated *Dclk1*^Cre/+^*;Apc*^fl/fl^ mice were provided drinking water containing 2.0%–2.5% DSS *ad libitum* for 5 days (days 19–24).

#### Therapeutic Treatment of *Apc*^min/+^ and *Dclk1*^Cre/+^*;Apc*^fl/fl^ Mice

PP (USP Rockville) was prepared at 5 mg/mL in HPMC-SV [(0.5% w/v) hydroxypropyl methyl-cellulose, 0.5% (v/v) benzyl alcohol, 0.4% (v/v) Tween 80]. Pyrvinium phosphate (PPh) was synthesized as described previously (25) and prepared at 1 mg/mL in HPMC-SV. ABT-263 (CAPOT Chemicals) was prepared at 10 mg/mL in HPMC-SV (solubilized by incubation in sonicating water bath at 45°C, 3 × 10 minutes). Sulindac (Sigma S8139-5G lot no. SLBF3303V0) was prepared at 6 mg/mL in captisol (captisol beta-cyclodextrin sulfobutyl ether 7 sodium salt; CyDex Inc CY-03A-199015). *Apc*^min/+^ and *Dclk1*^Cre/+^*;Apc*^fl/fl^ mice received 10 doses over 14 days of the following drugs or drug combinations: 25 mg/kg PP, 5 mg/kg PPh, 50 mg/kg ABT-263 either alone or in combination with 25 mg/kg PP or 5 mg/kg PPh, or a HPMC-SV vehicle control by oral gavage (0.1 mL); and 30 mg/kg sulindac either alone or in combination with 25 mg/kg PP, or vehicle control captisol by intraperitoneal injection (0.1 mL).

### Endoscopy

Adenoma onset, progression, and scoring in the distal colon were monitored by endoscopy as described previously (26).

### Tissue Collection and Histologic Analysis

Mouse colons were isolated by resection and opened longitudinally from the anus to the caecum, washed with PBS and fixed overnight in 10% neutral-buffered formalin and processed for analysis of adenoma number and area using ImageJ. Swiss rolls of the entire colon and colon tissue were prepared for histologic processing and hematoxylin and eosin (H&E) staining (26). X-Gal–stained tissue was counterstained with Nuclear fast red (Sigma-Aldrich, N3020). *Dclk1*^+^ cells in X-Gal–stained colonic tissue were identified using a rabbit polyclonal antibody against DCAMKL1 (Abcam, catalog no. ab31704, RRID:AB_873537, 1/1,000) using the DAB IHC staining kit (SK-4100, Vector Laboratories; Supplementary Fig. S1). Immune cells in adenomas were identified with anti-CD3 antibody (Abcam, catalog no. ab5690, RRID:AB_305055, 1/1,000) or anti-CD8 antibody (14-0808, e-Bioscience, 1/150) using the DAB IHC staining kit. Quantification of percent staining was done using Fiji-ImageJ analysis software (ImageJ, RRID:SCR_003070).

### Statistical Analysis

All data are representative of at least two independent experiments. Data are expressed as mean ± SEM (>5 mice/cohort). Comparisons between values from two groups were performed using the Student *t* test (unpaired with Welch correction) or Mann–Whitney test, as appropriate, provided by Prism 8.3.1 (PRISM, RRID:SCR_005375; GraphPad Prism, RRID:SCR_002798). *P* values < 0.05 were considered statistically significant. Power calculations were performed *post hoc* using the R statistical programming language and the software packages pwr (version 1.3–0) and effsize (version 0.8.1).

### Data Availability Statement

The data generated in this study are available within the article and its Supplementary Data files.

## Results

### Inflammation of Colonic Mucosa Promotes Adenoma Tumor Formation in *Apc*-Mutant Mice

#### Colon Adenoma Formation in *Apc*^min/+^ Mice

**FIGURE 1:**
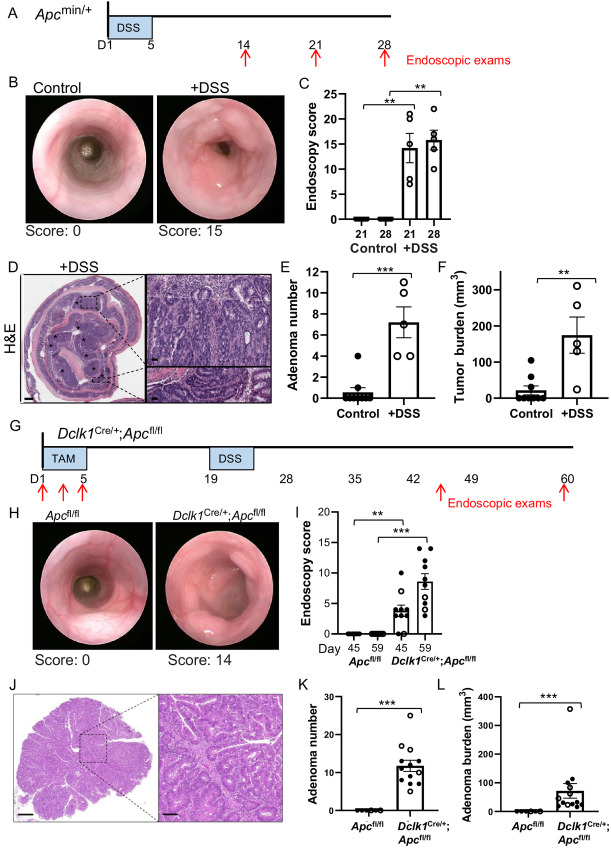
Acute inflammation promotes adenoma tumor development in *Apc*^min/+^ mice and in mice with Apc loss in *Dclk1*^+^ cells. **A,** Schematic representation of the DSS model. DSS was provided in the drinking water for 5 days, followed by normal drinking water. The formation of distal colonic adenomas is monitored by endoscopy at the indicated timepoints. **B,** Representative endoscopy images of distal colon adenoma tumors on day 21 of the DSS model for *Apc*^min/+^ mice given either normal drinking water (control) or DSS water (left). Endoscopy score is indicated below. **C,** Adenoma tumor burden of *Apc*^min/+^ mice treated with normal (control) and DSS drinking water was scored by endoscopy at days 21 and 28 of the DSS model; data are presented for individual mice with mean ± SEM shown (control *n* = 7, +DSS *n* = 5; ^**^P < 0.01, unpaired *t* test with Welch correction, power of 0.999 and 1.0 for days 21 and 28, respectively). **D,** Representative images of H&E-stained sections of *Apc*^min/+^ +DSS treated colon. The proximal colon is centered and the distal colon is along the outside edge. Adenomas are indicated (*). Enlarged images of adenoma tumors show moderate (top) and mild dysplasia (bottom). Enlarged, elongated nuclei, hyperchromatism, and disorganized crypts are present in adenomas. Scale bars, 440 μm; enlarged images 40 μm. Adenoma tumor number (**E**) and adenoma tumor burden (**F**) from individual mice treated with normal (control) and DSS drinking water taken at autopsy on day 28 of the DSS model. Data are presented for individual mice with mean ± SEM shown (control *n* = 9, +DSS *n* = 5; ^**^P < 0.005; ^***^P < 0.001, unpaired *t* test with Welch correction, power of 0.999 and 0.939 for **E** and **F**, respectively). **G,** Schematic representation of experimental set up for DSS treatment in *Dclk1*^Cre/+^;*Apc*^fl/fl^ mice [tamoxifen (Tam)/DSS model]. Tamoxifen was administered on days 1, 3, and 5 followed by DSS in the drinking water at day 19 for 5 days. The formation of distal colonic adenomas is monitored by endoscopy at the indicated timepoints. **H,** Representative endoscopy images of distal colon adenoma tumors on day 59 of the tamoxifen/DSS model for *Apc*^fl/fl^ or *Dclk1*^Cre/+^;*Apc*^fl/fl^ mice. Endoscopy score is indicated below. **I,** Adenoma tumor burden of *Apc*^fl/fl^ or *Dclk1*^Cre/+^;*Apc*^fl/fl^ mice was scored by endoscopy at days 45 and 59 of the tamoxifen/DSS model; data are presented for individual mice with mean ± SEM shown (*Apc*^fl/fl^*n* = 6, *Dclk1*^Cre/+^;*Apc*^fl/fl^*n* = 10; ^**^P < 0.01; ^***^P < 0.005, Mann–Whitney test, power of 0.818 and 0.997, days 45 and 59, respectively). **J,** Representative images of H&E-stained sections of *Dclk1*^Cre/+^;*Apc*^fl/fl^ adenomas, enlarged image shows dysplastic crypts (right). Adenoma tumor number (**K**) and adenoma tumor burden (**L**) from individual mice of the indicated genotypes taken at autopsy on day 60 of the tamoxifen/DSS model. Data are presented for individual mice with mean ± SEM shown (*Apc*^fl/fl^*n* = 6, *Dclk1*^Cre/+^;*Apc*^fl/fl^*n* = 13; ^***^P < 0.001; Mann–Whitney test, power of 0.999 and 0.428 for **K** and **L**, respectively). Closed symbols, female mice; open symbols, male mice.

*Apc* ^min/+^ mice are heterozygous for a truncating mutation in the *Apc* gene and as a result of spontaneous loss of heterozygosity of the remaining full-length wild-type allele, the mice develop extensive adenomatous polyps in the intestine (7). While numerous polyps develop in the small intestine, very few polyps occur in the colon. We found that *Apc*^min/+^ mice developed anemia, presumably as a result of the intestinal tumor burden (8) and died between 13 and 16 weeks of age (mean age 101.9 ± 2.8 days, *n* = 48; Supplementary Fig. S2A). At this stage, 54% of mice developed an adenoma in the colon with no adenomas observed in the colons of the remaining mice (Supplementary Fig. S2A). Treating the mutant mice (6–8 weeks of age) with DSS promotes inflammation of the colonic mucosa and increases tumor burden in the colon as previously shown (15). To confirm that *Apc*^min/+^ mice develop increased adenoma burden with DSS treatment, *Apc*^min/+^ mice were exposed to DSS (Fig. 1A for treatment protocol). We detected profound increases in adenoma formation, both in number and size, in the distal and middle colons, but not the proximal colon (Supplementary Fig. S2B, bottom). Endoscopy revealed the presence of multiple lesions at day 21 and day 28 after DSS exposure (Fig. 1B and C). Low- and moderate-grade dysplasia was present in the adenomas from the DSS-treated mice characterized by enlarged elongated nuclei and hyperchromatism and disorganization of colonic crypts (Fig. 1D). As expected, adenomas were almost completely absent in treatment-naïve *Apc*^min/+^ mice (9–11 weeks of age) compared with large numbers of adenomas in the distal and middle colons in the age-matched DSS-treated mice (Fig. 1E and F).

#### Colon Adenoma Formation in *Dclk1*^C^^re^^/+^;*Apc*^fl/fl^ Mice

We used a second inflammation-induced colon adenoma model in which *Apc* truncation is driven by Cre-mediated recombination of the *Apc* allele (*Apc*^fl/fl^) in *Dclk1^+^* cells. Expansion of *Dclk1*^+^ cells occurs following DSS-induced colitis and conditional recombination of the *Apc*^fl^ allele in an expanded population of *Dclk1*^+^ cells leads to adenomas in the colon as reported previously (10). We confirmed that acute exposure to DSS following tamoxifen-induced Cre-mediated recombination of the *Apc* gene *Dclk1*^Cre/+^*;Apc*^fl/fl^ mice results in multiple adenomas in the distal and middle colon (Fig. 1G; Supplementary Fig. S2C). Endoscopies of *Dclk1*^Cre/+^*;Apc*^fl/fl^ treated with tamoxifen and DSS shows the presence of multiple adenomas whereas tumors were completely absent in *Apc*^fl/fl^ littermates (Fig. 1H and I) and prior to DSS treatment (Supplementary Fig. S2D). Low- and moderate-grade dysplasia was present in the adenomas from the DSS-treated mice (Fig. 1J). Large adenomas were present in the distal and middle colons of all of the DSS-treated *Dclk1*^Cre/+^*;Apc*^fl/fl^ mice (Fig. 1K and L). The increased colon tumor formation in DSS-treated *Apc*^min/+^ and *Dclk1*^Cre/+^*;Apc*^fl/fl^ cohorts was associated with substantial mortality as the large tumor burden required the mice to be euthanized 2 and 4 weeks after treatment, respectively. We conclude that DSS-induced colon adenoma formation in both *Apc*^min/+^ and *Dclk1*^Cre/+^*;Apc*^fl/fl^ mice represent reliable models of human colorectal adenomas formation, as shown in previous studies (10, 15), and the adenoma burden is sufficient for investigating the effects of candidate drugs.

### Effects of Wnt Pathway and Bcl-2 Inhibition on Colon Tumorigenesis

**FIGURE 2:**
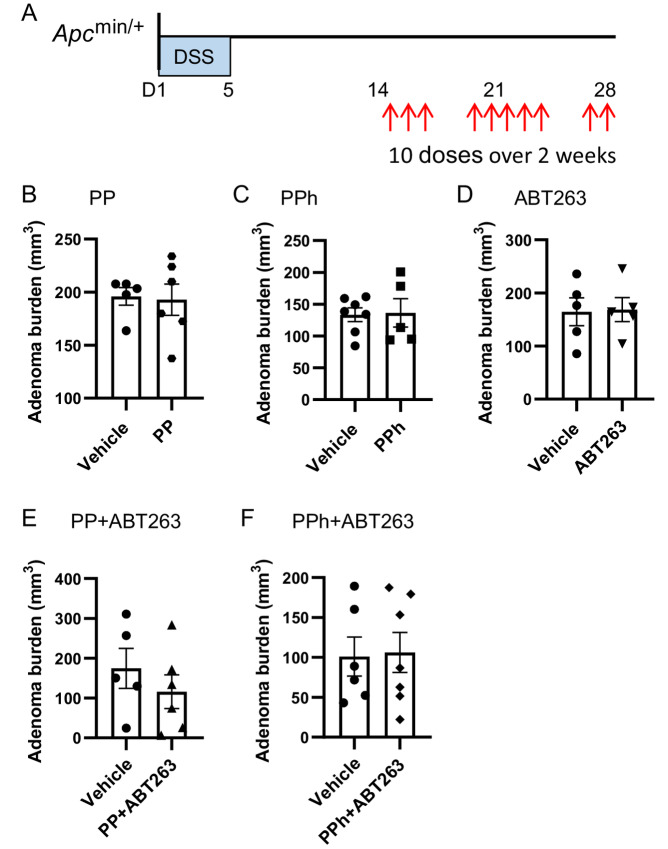
Treatment with Wnt pathway and proapoptotic compounds does not reduce colon adenoma burden in *Apc*^min/+^ mice. **A,** Schematic representation of the DSS model and treatment: *Apc*^min/+^ mice with established colon adenomas were treated with inhibitor compounds or a vehicle control, 10 doses over 2 weeks. Drug treatment commenced at 14 days after DSS treatment. **B–F,** Colon adenoma burden was scored following treatment: **B,** vehicle control (*n* = 5) or Wnt pathway inhibitor, PP (*n* = 6); **C,** vehicle control (*n* = 7) or PPh (*n* = 5); **D,** vehicle control or proapoptotic Bcl-2 inhibitor, ABT263 (*n* = 5 per cohort); **E,** vehicle control (*n* = 5) or PP in combination with ABT263 (PP+ABT263) (*n* = 6); **F,** vehicle control (*n* = 6) or PPh+ABT263 (*n* = 7). Data are presented for individual mice with mean ± SEM shown (*n* > 5 per cohort as indicated; unpaired *t* test, one-tail, power of 0.053, 0.052, 0.051, 0.128, and 0.052 for **B–F**, respectively).

We have shown that cytotoxic activity of a Wnt pathway inhibitor PP is more effective in combination with a pro-apoptotic Bcl-2 inhibitor (ABT737/ABT263) in killing colorectal cancer cells *in vitro* and in a xenografted human colon tumor *in vivo* (27). To determine whether the combination of PP and ABT263 was also effective in killing colon adenoma cells *in vivo*, we treated *Apc*^min/+^ mice with PP, an orally available derivative of PP, PPh, or ABT263 alone and in combination (Fig. 2A). We used the DSS treatment (Fig. 1) to promote colon tumorigenesis in *Apc*^min/+^ mice. *Apc*^min/+^ mice were treated with 10 doses of drug from day 14 (i.e., 9 days after DSS treatment had finished; Fig. 2A). Adenomas were measured to assess overall tumor burden. There was no significant reduction in the adenoma burden in mice treated either with PP, PPh, or ABT263 alone or with the combination of PP/PPh+ATB263 (Fig. 2B–F), although the mean adenoma burden was slightly reduced with the PP+ABT263 combination. While the orally available PPh was effective in reducing colon tumor xenografts *in vivo* (27), there was no difference in adenoma burden in mice treated with PP or PPh, either alone or in combination with ABT263. It is possible that longer treatments might be needed to reduce the growth of adenomas with these drug combinations.

### Wnt Inhibition in Combination with NSAID Therapy Suppresses Colon Adenoma Growth

**FIGURE 3:**
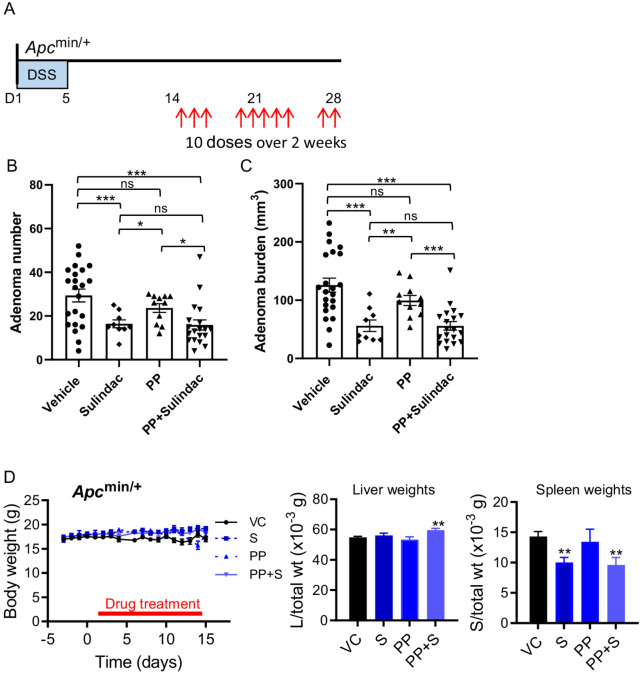
Combination treatment with Wnt pathway inhibition and NSAID reduces colon tumorigenesis in *Apc*^min/+^ mice. **A,***Apc*^min/+^ mice with established colon adenomas were treated with inhibitor compounds or a vehicle control, 10 doses over 2 weeks. Drug treatment commenced at 14 days after DSS treatment. Total colon adenoma numbers (**B**) and adenoma burden (**C**) in vehicle-(*n* = 22), sulindac-(*n* = 9), PP-(*n* = 11), or sulindac+PP-(*n* = 19) treated *Apc*^min/+^ mice with established colon adenomas. Shown are mean ± SEM (*n* > 9) per cohort as indicated. In **B**, Student unpaired *t* tests were applied to test for significant differences in adenoma number between vehicle versus sulindac (power 0.753), vehicle versus PP (power 0.245), vehicle versus PP+sulindac (power 0.932), sulindac versus PP (power 0.700), sulindac versus PP+ sulindac (power 0.052), and PP versus PP+sulindac (power 0.586). In **C**, Mann–Whitney tests were applied to test for significant differences in colon adenoma burden between vehicle versus sulindac (power 0.920), vehicle versus PP (power 0.292), vehicle versus PP+sulindac (power 0.996), sulindac versus PP (power 0.881), sulindac versus PP+ sulindac (power 0.0.05), and PP versus PP+sulindac (power 0.948). Significant differences are indicated (^***^P < 0.001; ^**^P < 0.01; ^*^P < 0.05; ns, not significant). **D,** Body weight over treatment period (indicated in red) showing weights from 4 days prior to treatment to the end of experiment (left), liver and spleen weights relative to total body weight (middle and right). VC, vehicle control. Shown are mean ± SEM (*n* > 9 per cohort as indicated in above; ^**^P < 0.01, unpaired *t* test).

While we observed little effect of PP on adenoma growth, we investigated its activity in combination with the NSAID sulindac in the *Apc* DSS models. Sulindac has been used extensively in the clinic and has been shown to confer a therapeutic benefit for patients with colorectal cancer and in a reduction of adenomas in patients with familial adenomatous polyposis (28–30). Both adenoma number and burden were significantly reduced in DSS-treated *Apc*^min/+^ mice following treatment with sulindac or when sulindac was used in combination with PP compared with vehicle-treated or PP-treated animals (10 doses over 14 days; Fig. 3A–C). However, there was no significant difference between sulindac alone and sulindac+PP suggesting that the addition of PP does not provide any further benefit in *Apc*^min/+^ mice. There was no evident toxicity among the treatment groups, as body weights were not significantly changed prior to, at the start and end of drug treatments, although weights were slightly higher (<10% change) in all treatment groups compared with vehicle control (Fig. 3D). In addition, liver to body weight ratios were not significantly altered in sulindac and PP treatments, indicating minimal toxicity. Indeed, the ratios were increased slightly (7.9%) in the combination treatments (Fig. 3D). Spleen weights relative to total body weights were decreased in sulindac and sulindac plus PP treatments compared with vehicle (Fig. 3D). Depending on their age *Apc*^min/+^ mice tend to develop anemia and a consequently, a larger spleen than normal mice. Treatment of *Apc*^min/+^ mice with sulindac reduces the spleen weight back toward its normal weight (14).

**FIGURE 4:**
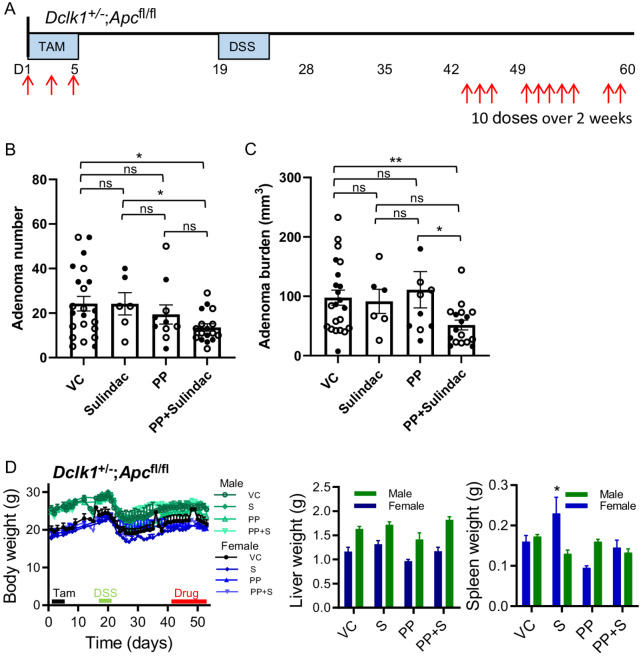
Combination treatment with Wnt pathway inhibition and NSAID reduces colon tumorigenesis in *Dclk1*^Cre±^;*Apc*^fl/fl^ mice. **A,***Dclk1*^Cre/+^;*Apc*^fl/fl^ mice with established colon adenomas were treated with inhibitor compounds with 10 doses over 14 days. Treatment commenced at day 38 following tamoxifen treatment. Total colon adenoma numbers (**B**) and adenoma burden (**C**) in vehicle-(VC; *n* = 22), sulindac-(*n* = 6), PP-(*n* = 10), or sulindac+PP-(*n* = 17) treated mice. Shown are mean ± SEM (*n* > 6 per cohort as indicated). Mann–Whitney tests were applied to test for significance in: colon adenoma number between vehicle versus sulindac (power 0.05), vehicle versus PP (power 0.131), vehicle versus PP+sulindac (power 0.730), sulindac versus PP (power 0.101), sulindac versus PP+ sulindac (power 0.709), and PP versus PP+sulindac (power 0.300; **B**); colon adenoma burden between vehicle versus sulindac (power 0.055), vehicle versus PP (power 0.075), vehicle versus PP+sulindac (power 0.789), sulindac versus PP (power 0.071), sulindac versus PP+ sulindac (power 0.553), and PP versus PP+sulindac (power 0.607; **C**). Significant differences are indicated ^**^P < 0.01; ^*^P < 0.05; ns, not significant. **D,** Body weights for male and female *Dclk1*^Cre/+^;*Apc*^fl/fl^ mice over the course of the experiment (left), liver and spleen weights relative to total body weight (middle and right). Shown are mean ± SEM [male: VC *n* = 13, sulindac *n* = 4, PP *n* = 5, *n* = 10); female: VC *n* = 9, sulindac *n* = 2, PP *n* = 5, PP+sulindac *n* = 7; ^**^P < 0.05 (*n* = 2), unpaired *t* test]. Closed symbols, female mice; open symbols, male mice.

We did not see a significant effect of sulindac or PP treatment alone in DSS-treated *Dclk1*^Cre/+^*;Apc*^fl/fl^ mice (10 doses over 14 days; Fig. 4A–C). However, the combination of sulindac with PP resulted in a significant reduction in both adenoma number and adenoma burden in DSS-treated *Dclk1*^Cre/+^*;Apc*^fl/fl^ mice compared with vehicle-treated mice (Fig. 4B,C). There was a significant reduction in adenoma number with the combination compared with sulindac alone but not in comparison with PP alone where there was a modest but nonsignificant reduction compared with vehicle (Fig. 4B). Adenoma burden was significantly reduced with the combination of sulindac and PP compared with PP alone but not sulindac alone (Fig. 4C). Male and female body and liver weights were not significantly different in treatment groups compared with vehicle-treated *Dclk1*^Cre/+^*;Apc*^fl/fl^ mice (Fig. 4D). PP treatment did result in an increase in spleen weights of female (*P* = 0.020) but not male mice and there was no change with sulindac alone or in combination with PP (Fig. 4D). Histologic analysis demonstrates dysplasia in the adenomas, as shown in Fig. 1, and nuclear and cytoplasmic β-catenin staining in vehicle control tissue (Supplementary Fig. S3S). In the drug treatment groups, there is increased cytoplasmic retention of β-catenin (Supplementary Fig. S3S), indicating that nuclear accumulation of β-catenin is blocked by sulindac, PP, and the combination treatment, consistent with previous reports (31, 32). Although adenomas were reduced with sulindac alone and PP+sulindac in the *Apc*^min/+^ mice, these results indicate that a combination of anti-inflammatory treatment with a Wnt pathway inhibitor provides an opportunity for reducing tumor number and burden in *Apc*-defective colon adenoma cells.

### Tumor Immune Cell Infiltration Following Wnt Inhibition

**FIGURE 5:**
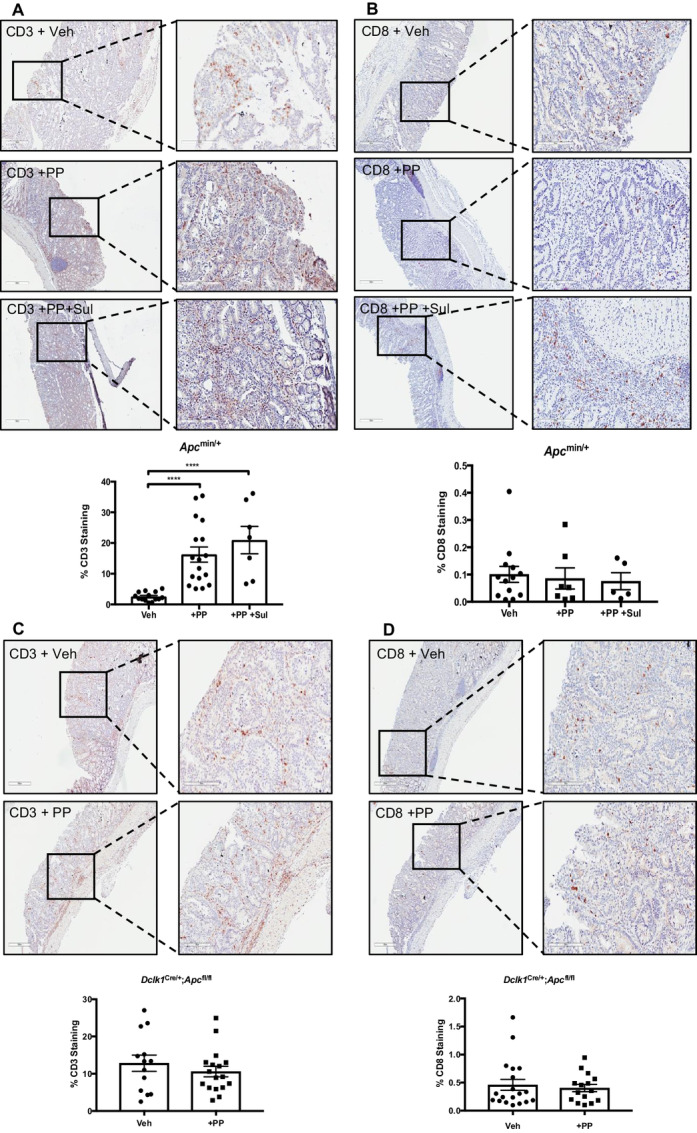
Tumor–immune cell infiltration in inflammation-induced adenomas following Wnt inhibition. IHC staining of vehicle (*n* = 3), PP (*n* = 5), and PP+sulindac (*n* = 3) treatment in DSS-induced adenomas in *Apc*^min/+^ mice (**A** and **B**) and vehicle (*n* = 4) and PP (*n* = 3) treatment in DSS-induced adenomas in *Dclk1*^Cre/+^;*Apc*^fl/fl^ mice (**C** and **D**). Tissue sections were immunostained with anti-CD3 (**A** and **C**) and anti-CD8 antibodies (**B** and **D**). Quantification is shown in the plots below as percent positively stained cells. Each dot represents one tumor, shown are mean ± SEM (*n* > 2; ^****^P < 0.001, unpaired *t* test).

Oncogenic Wnt/β-catenin signaling has been shown to reduce T-cell recruitment into the tumor microenvironment (33, 34). We therefore asked whether treatment with a Wnt inhibitor would increase tumor immune cell infiltration. Indeed, we detected a significant increase of CD3^+^ immune cells after PP treatment in tumors of *Apc*^min/+^ mice compared with vehicle-treated mice (Fig. 5A). However, there was no increase in CD8^+^ cytotoxic T cells (Fig. 5B) which suggests that the majority of infiltrating CD3^+^ immune cells are CD4^+^ Th cells or regulatory T cells. Additional treatment with sulindac did not change the number of infiltrating immune cells suggesting that the increase is Wnt pathway dependent. In contrast, there was no increase in CD3^+^ immune cells in colon adenomas of *Dclk1*^Cre/+^;*Apc*^fl/fl^ mice after treatment with PP, even though these tumors had overall higher numbers of CD3^+^ immune cells (Fig. 5C). Similar to the tumors from *Apc*^min/+^ mice, there was no difference in the numbers of cytotoxic CD8^+^ T cells in tumors from *Dclk1*^Cre/+^;*Apc*^fl/fl^ mice (Fig. 5D). These data suggest that the increased immune cell infiltration after treatment with PP is not due to tumor cell intrinsic Wnt signaling inhibition but rather due to the reversal of the lymphodepletion phenotype developed in *Apc*^min/+^ mice, most likely secondary to LOH-induced intestinal tumorigenesis, and which is not observed in *Apc*^fl/fl^ mice (17, 35). Consequently, the reduction in tumor burden by PP/sulindac treatment in the *Dclk1*^Cre/+^;*Apc*^fl/fl^ mice is clearly independent of tumor infiltration of CD3 T cells and must be driven by a currently unknown mechanism.

## Discussion

Colonoscopic detection of adenomas has helped to reduce the number of colorectal cancer cases in the community; however, it is a costly intervention (36) and more people are dying of the disease every year (37). More than 90% of precancerous lesions in the colon involve either mutations to the *APC* or *BRAF* genes; drugs capable of specifically killing cells with these mutations should be useful for reducing the incidence of colon cancer. Trials aimed at the chemoprevention of colon cancer in the general population have been limited, for example, aspirin (38) or celecoxib (39) and the results have been disappointing. Presumably this is because single anti-inflammatory agents reduce proliferation of adenoma cells, but do not kill them.

We have discovered that the combination of a Wnt inhibitor (PP/PPh) with a Bcl-2-family inhibitor (ABT263) is effective in killing colorectal cancer cells with *APC* mutations both *in vitro* and in colon tumor xenografts (27). From our results on two Apc mouse models, colon adenomas were not significantly reduced by PP and ABT263 either when used singly or in combination. The activities of Bcl-2 and Bcl-XL are important in tumor development (40, 41), so it was surprising that we did not see an effect with ABT263 in our Apc inflammation–driven models. The antitumor effect of PP+Bcl-2 inhibition may be restricted to later invasive/metastatic stages of tumorigenesis. It is also possible that other Bcl-2 family members such as Bcl-XL or Bcl-W are upregulated in the adenomas, rendering ABT263 less effective, given that Bcl-2 is the critical ABT-263 target *in vivo* (42). Indeed recent studies show that BCL-2 is downregulated upon APC mutation and inhibition of Bcl-XL was shown to impair adenoma outgrowth, although BCL-XL inhibition was not effective on preexisting adenomas (43). Our results also imply that differences in drug response may be attributable to differences in the immune milieu which is vastly different in the inflammatory-driven *Apc*-mutant model to that of xenografted nude mice.

When PP was combined with sulindac, there was a significant reduction in colon adenoma formation and growth in the *Dclk1*^Cre/+^;*Apc*^fl/fl^ model. As expected sulindac decreased adenomas in the *Apc*^min/+^ model (13, 14), but in the *Apc*^min/+^ mice there was no further increase in the killing of the adenomas when PP was added to the sulindac. Likewise, sulindac treatment alone did not effect *Dclk1*^Cre/+^;*Apc*^fl/fl^ adenomas. It was surprising that we did not see an effect of PP treatment in our experiments. It is possible that sufficient concentrations of the PP were not were not present in the colon mucosa or that the treatment time needed to be extended. As shown previously (30), drug treatments resulted in retention of cytoplasmic β-catenin, suggesting that sulindac and PP act to block β-catenin from the nucleus, but increased cytoplasmic β-catenin indicates the protein is not efficiently targeted for degradation in colon adenoma cells. Nevertheless, our experiments in the *Dclk1*^Cre/+^;*Apc*^fl/fl^ mice showed a benefit with the PP+sulindac combination. Our experiments highlight a different treatment response sensitivity between mice with a systemic Apc mutation or a conditional mutant and underscore the importance of investigating more than one model system/mechanism of tumorigenesis. While our studies were focused on the colon, there is evidence to suggest that treatment with the agents used here could be effective in reducing small intestinal tumors (41, 44, 45).

We used acute inflammation induced by DSS to promote colon tumorigenesis in both *Apc*^min/+^ and in *Dclk1*^Cre/+^*;Apc*^fl/fl^ mice. While *Apc*^min/+^ mice are well characterized as a model for the human FAP, the mice spontaneously develop tumors in the small intestine but not in the colon. The development of few (<1 colon adenoma/mouse) in our *Apc*^min/+^ mice precluded assessment of drug treatments on colon adenoma formation. Even the *Apc*^+/MinFCCC^ mouse model which develops multiple colon adenomas, the mean number of adenomas is only 3.7 ± 0.3 (45). We therefore used acute DSS exposure which has been shown to promote the growth of early colonic cryptal lesions, dysplastic aberrant crypt foci or adenomatous lesions that resulted in high numbers of large adenomas in the colon (15). In both *Apc*^min/+^ and in *Dclk1*^Cre/+^*;Apc*^fl/fl^ models, there were significant increases in the numbers of colon adenomas 4 weeks after the DSS treatment. The action of sulindac may have been via antagonism of inflammatory cytokines elicited by DSS that resulted in reduced growth, although the effects of sulindac on intestinal adenomas is reported even in the absence of inflammatory stimuli (13, 14). Moreover, sulindac treatment is reported to result in reduced β-catenin expression and Wnt signaling in mouse and human *APC*-mutant adenomas (14, 31). In the *Dclk1*^Cre/+^*;Apc*^fl/fl^ mice, DSS was used to activate the expression of *Dclk1*^+^ cells in which Apc was truncated. Lineage tracing experiments show a proportion of *Dclk1*^+^ cells also express Lrg5 and while reported to represent a separate population to Lgr5-expressing intestinal stem cells, nevertheless expand during injury and early tumorigenesis (46). While loss of Apc in Lgr5^+^ cells (4) leads to tumorigenesis, we found that the growth and number of adenomas was more robust in the *Dclk1*^+^/DSS model due to the mosaic pattern and low levels of expression in the colon of Lgr5-CreERT2 mice (47). The further reduction in adenoma number with the sulindac+PP combination compared with sulindac alone suggests an increased sensitivity of the *Dclk1*^+^ cell–induced *Apc*-mutant adenoma cells compared with the adenoma cells in the constitutive *Apc*^min/+^ model. It will be important to determine whether these drugs exert similar effects in a stem cell Apc tumor model without DSS, such as Lrig1-CreERT;*Apc*^fl/+^ mice (48).

The *Dclk1*^Cre/+^*;Apc*^fl/fl^ adenomas were not as sensitive to sulindac (as a single agent) as the *Apc*^min/+^ adenomas; however, the sulindac+PP combination treatment reduced the number and burden of the adenomas when compared with untreated mice. There was a reduction in adenoma number with the combination compared with sulindac alone and a reduction in burden compared with PP alone. The DSS inflammation, which stimulates the Dclk-1 cells from quiescence, likely leads to the activation of Wnt production which must be important for the proliferation of the Dclk-1 generated adenoma stem cells. The increased CD3^+^ immune cells we observed in *Apc*^min/+^ adenomas following Wnt inhibition suggest that the block in immune infiltration upon activated oncogenic Wnt signaling can be reversed. However, there was no change in immune cell infiltration in *Dclk1*^Cre/+^*;Apc*^fl/fl^ adenomas following Wnt inhibition albeit with higher baseline levels of CD3^+^ cells. These data reveal differences between the two colorectal cancer models regarding the susceptibility of immune cell infiltration of adenomas to inhibition of Wnt signaling. *Apc*^Min^ mice with intestinal adenomas develop splenomegaly associated with lymphodepletion of CD3, CD4, and CD8 T cells and natural killer cells (17, 35). We cannot therefore rule out the possibility that the observed increase of CD3 infiltration in *Apc*^Min^ adenomas is due to a Wnt-dependent reversal of the immune cell loss in the spleen rather than an increase in the ability of *Apc*-mutant immune cells to mobilize to the tumor after Wnt inhibition. It will be interesting to further assess neutrophilic and macrophage infiltration, in addition to specific immunophenotyping of T cells and their subsets, in tumors in future work.

Our results suggest that PP in combination with sulindac could be considered for reducing the colon adenoma burden in the community. The oral administration of the PP/sulindac combination should be safe; however, it would be appropriate to search for other inhibitors which interfere more specifically with the signaling systems activated when Apc is truncated, for example, CK1α (11). It will also be important to test other proapoptotic agents, for example, Bcl-XL- or Mcl-1–specific inhibitors (49, 50) for their potency for killing adenomas in the presence of sulindac or PP, and whether those drugs might enhance the reduction of colon adenomas by the combined PP/sulindac treatment.

## Supplementary Material

Figure S1 Xgal and DCLK1 staining of colon crypts from DCLK1-CreERT2 BAC transgenic mice

Figure S2 Adenoma tumour development in Apcmin/+ and Dclk1Cre/+;Apcfl/fl mice

Figure S3 Histology and immunohistochemical analysis of beta-catenin in adenomas from treated Dclk1Cre/+;Apcfl/fl mice.
