# Supplementary material for: Combined Treatment with a WNT Inhibitor and the NSAID Sulindac Reduces Colon Adenoma Burden in Mice with Truncated APC
Source: Cancer Res Commun. 2022 Feb 2;2(2):66–77. doi: 10.1158/2767-9764.CRC-21-0105 (PMC9973414; doi:10.1158/2767-9764.CRC-21-0105)
Supplement: Figure S1 — Xgal and DCLK1 staining of colon crypts from DCLK1-CreERT2 BAC transgenic mice [file crc-21-0105-s01.pptx]

## Slide 1
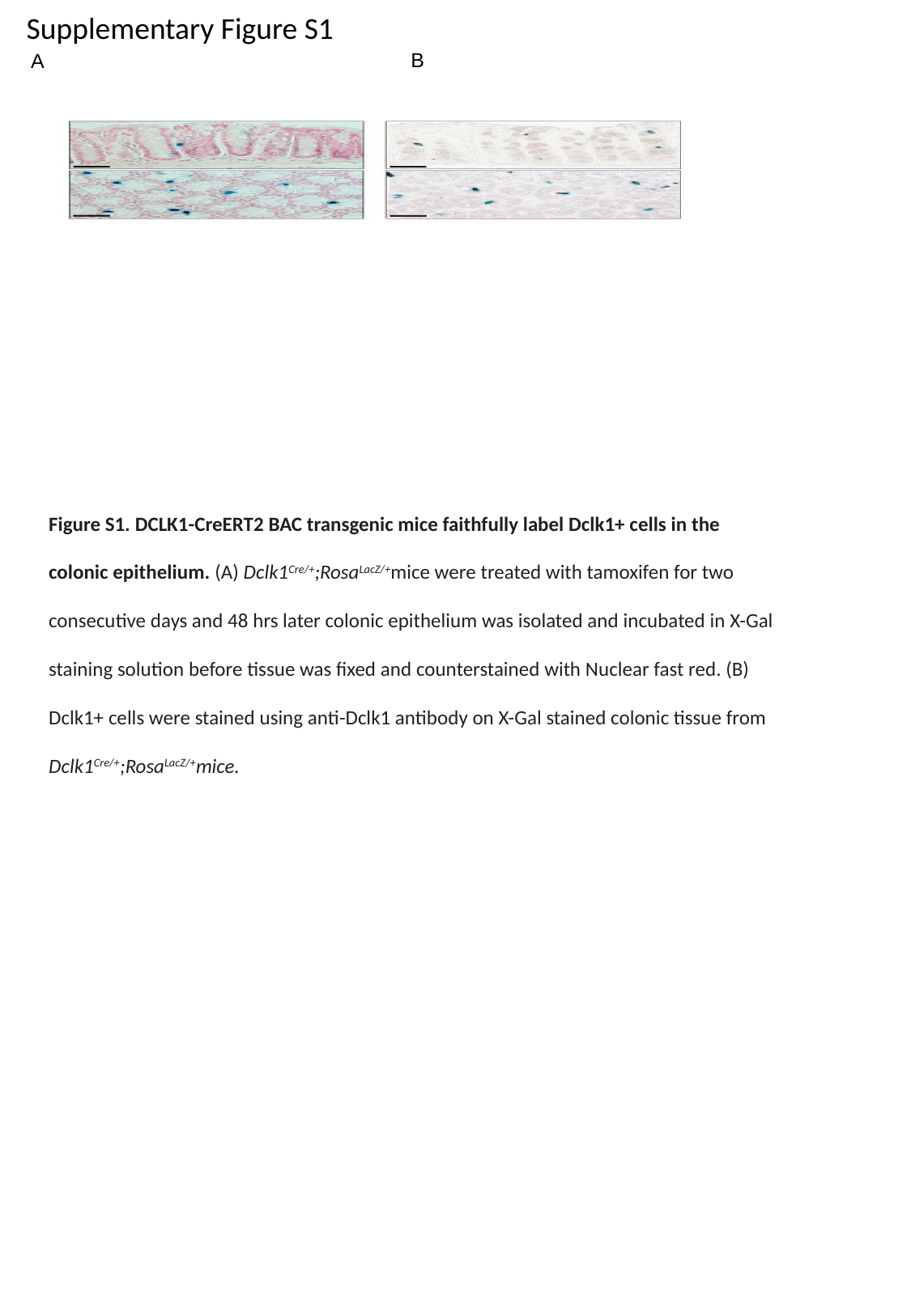

Supplementary Figure S1
B
A
Figure S1. DCLK1-CreERT2 BAC transgenic mice faithfully label Dclk1+ cells in the colonic epithelium. (A) Dclk1Cre/+;RosaLacZ/+mice were treated with tamoxifen for two consecutive days and 48 hrs later colonic epithelium was isolated and incubated in X-Gal staining solution before tissue was fixed and counterstained with Nuclear fast red. (B) Dclk1+ cells were stained using anti-Dclk1 antibody on X-Gal stained colonic tissue from Dclk1Cre/+;RosaLacZ/+mice.
