# Supplementary material for: Combined Treatment with a WNT Inhibitor and the NSAID Sulindac Reduces Colon Adenoma Burden in Mice with Truncated APC
Source: Cancer Res Commun. 2022 Feb 2;2(2):66–77. doi: 10.1158/2767-9764.CRC-21-0105 (PMC9973414; doi:10.1158/2767-9764.CRC-21-0105)
Supplement: Figure S2 — Adenoma tumour development in Apcmin/+ and Dclk1Cre/+;Apcfl/fl mice [file crc-21-0105-s02.pptx]

## Slide 1
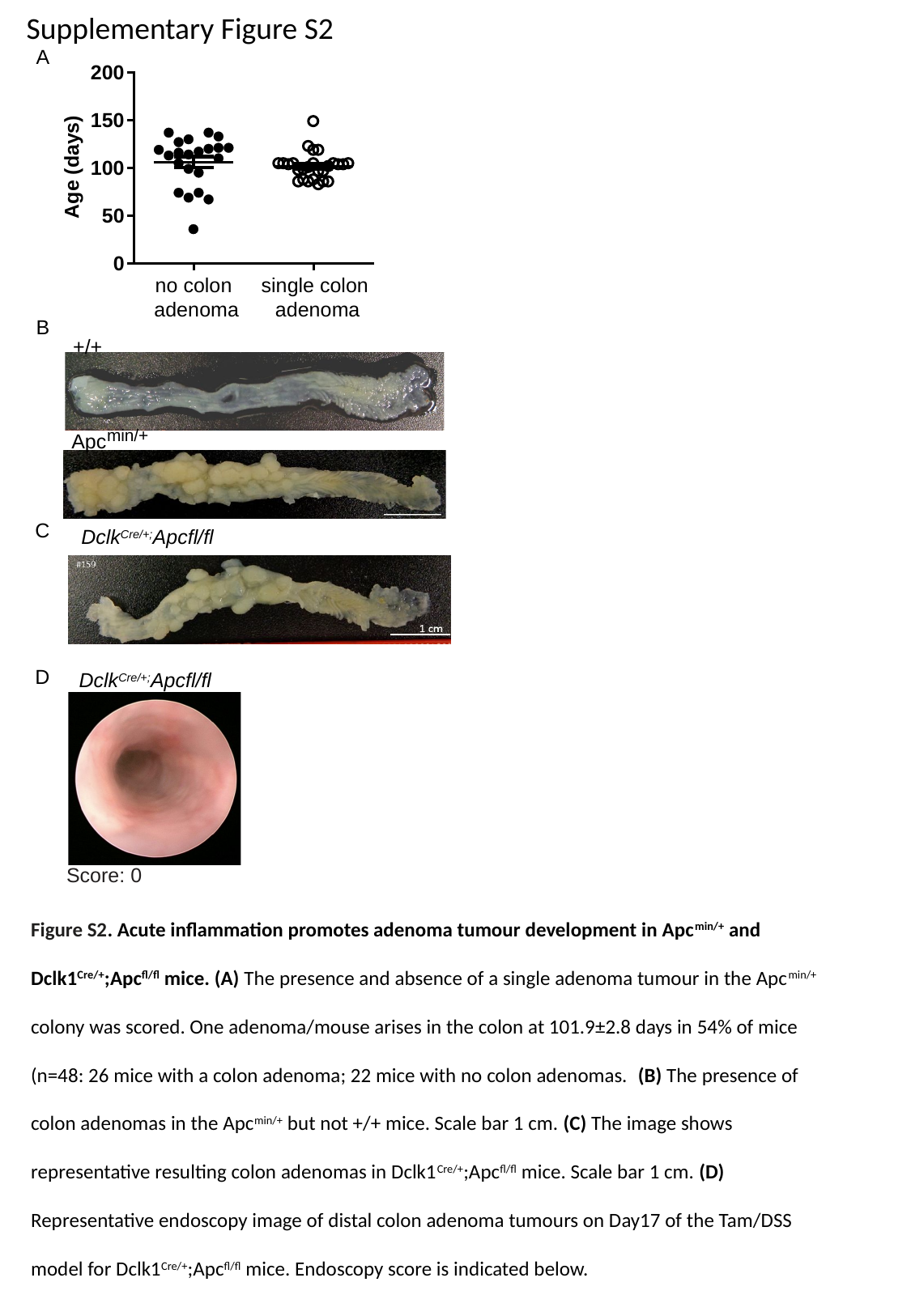

Supplementary Figure S2
A
no colon
adenoma
single colon
adenoma
B
+/+
min/+
Apc
C
DclkCre/+;Apcfl/fl
D
DclkCre/+;Apcfl/fl
Score: 0
Figure S2. Acute inflammation promotes adenoma tumour development in Apcmin/+ and Dclk1Cre/+;Apcfl/fl mice. (A) The presence and absence of a single adenoma tumour in the Apcmin/+ colony was scored. One adenoma/mouse arises in the colon at 101.9±2.8 days in 54% of mice (n=48: 26 mice with a colon adenoma; 22 mice with no colon adenomas. (B) The presence of colon adenomas in the Apcmin/+ but not +/+ mice. Scale bar 1 cm. (C) The image shows representative resulting colon adenomas in Dclk1Cre/+;Apcfl/fl mice. Scale bar 1 cm. (D) Representative endoscopy image of distal colon adenoma tumours on Day17 of the Tam/DSS model for Dclk1Cre/+;Apcfl/fl mice. Endoscopy score is indicated below.
