# Supplementary material for: Combined Treatment with a WNT Inhibitor and the NSAID Sulindac Reduces Colon Adenoma Burden in Mice with Truncated APC
Source: Cancer Res Commun. 2022 Feb 2;2(2):66–77. doi: 10.1158/2767-9764.CRC-21-0105 (PMC9973414; doi:10.1158/2767-9764.CRC-21-0105)
Supplement: Figure S3 — Histology and immunohistochemical analysis of beta-catenin in adenomas from treated Dclk1Cre/+;Apcfl/fl mice. [file crc-21-0105-s03.pptx]

## Slide 1
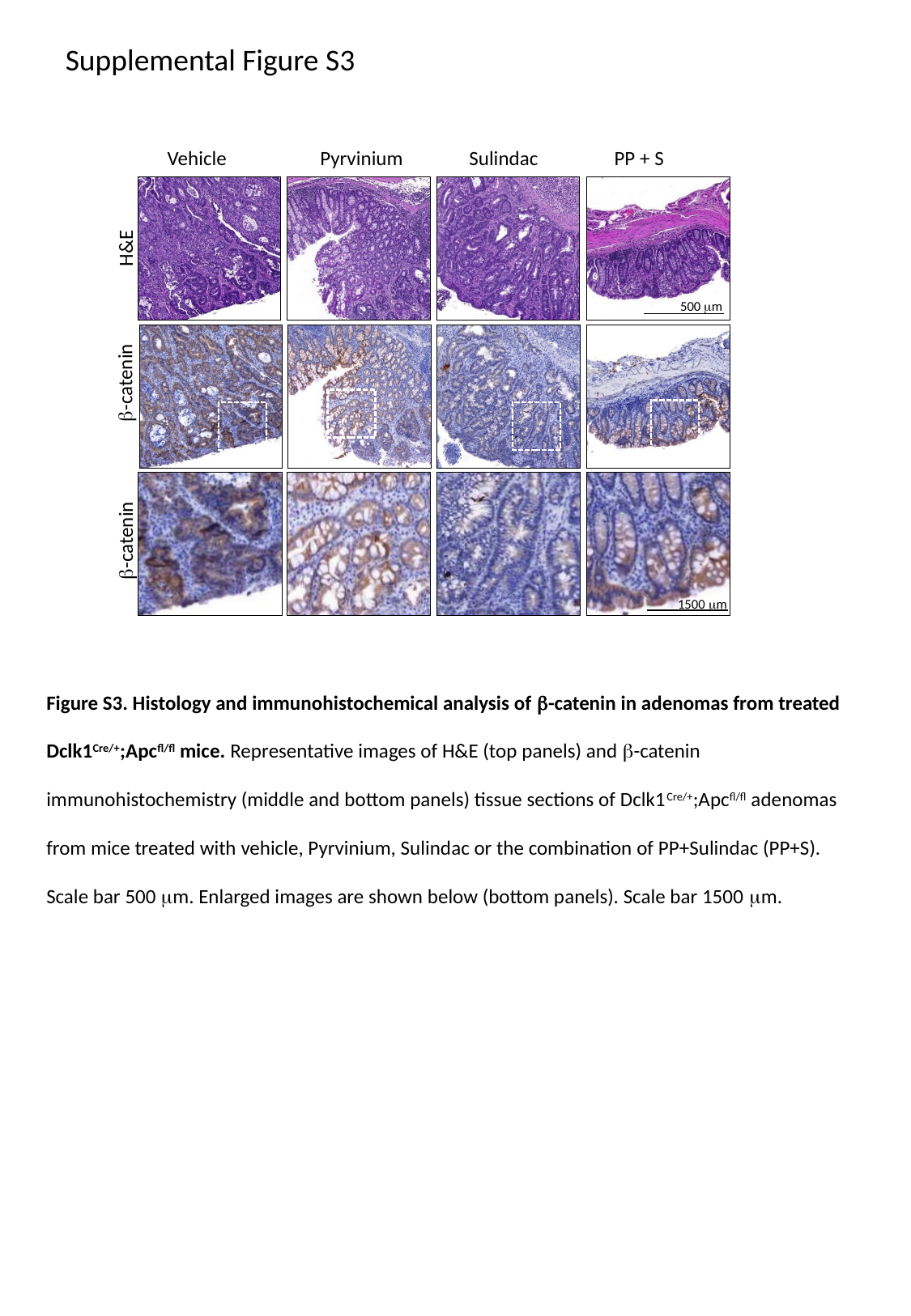

Supplemental Figure S3
Vehicle
Pyrvinium
Sulindac
PP + S
H&E
500 mm
b-catenin
b-catenin
1500 mm
Figure S3. Histology and immunohistochemical analysis of b-catenin in adenomas from treated Dclk1Cre/+;Apcfl/fl mice. Representative images of H&E (top panels) and b-catenin immunohistochemistry (middle and bottom panels) tissue sections of Dclk1Cre/+;Apcfl/fl adenomas from mice treated with vehicle, Pyrvinium, Sulindac or the combination of PP+Sulindac (PP+S). Scale bar 500 mm. Enlarged images are shown below (bottom panels). Scale bar 1500 mm.
